# The Impact of Weight Loss on Inflammation, Oxidative Stress, and Mitochondrial Function in Subjects with Obesity

**DOI:** 10.3390/antiox13070870

**Published:** 2024-07-19

**Authors:** Neus Bosch-Sierra, Carmen Grau-del Valle, Jonathan Hermenejildo, Alberto Hermo-Argibay, Juan Diego Salazar, Marta Garrido, Beatriz Navajas-Porras, Guillermo Sáez, Carlos Morillas, Celia Bañuls

**Affiliations:** 1 Department of Endocrinology and Nutrition, University Hospital Doctor Peset, Foundation for the Promotion of Health and Biomedical Research in the Valencian Region (FISABIO), 46017 Valencia, Spain; neus.bosch@fisabio.es (N.B.-S.); graudel89@gmail.com (C.G.-d.V.); jonathan.hermnejildo@fisabio.es (J.H.); alberto.hermo@fisabio.es (A.H.-A.); mdjuansalazar@gmail.com (J.D.S.); martagabau@gmail.com (M.G.); beatriz.navajas@fisabio.es (B.N.-P.); carlos.morillas@uv.es (C.M.); 2 Service of Clinical Analysis, University Hospital Dr. Peset, Foundation for the Promotion of Health and Biomedical Research in the Valencian Region (FISABIO), 46017 Valencia, Spain; guillermo.saez@uv.es; 3 Department of Biochemistry and Molecular Biology, Faculty of Medicine and Odontology, University of Valencia, 46010 Valencia, Spain; 4 Department of Medicine, Faculty of Medicine and Odontology, University of Valencia, 46010 Valencia, Spain

**Keywords:** obesity, oxidative stress, mitochondrial respiration, mitochondrial dysfunction, weight loss, inflammation

## Abstract

Inflammation, oxidative stress, and mitochondrial function are implicated in the development of obesity and its comorbidities. The purpose of this study was to assess the impact of weight loss through calorie restriction on the metabolic profile, inflammatory and oxidative stress parameters, and mitochondrial respiration in an obese population. A total of 109 subjects underwent two cycles of a very low-calorie diet alternated with a low-calorie diet (24 weeks). We analyzed biochemical and inflammatory parameters in serum, as well as oxidative stress markers, mRNA antioxidant gene expression, and mitochondrial respiration in peripheral blood mononuclear cells (PBMCs). After the intervention, there was an improvement in both insulin resistance and lipid profiles, including cholesterol subfractions. Weight loss produced a significant reduction in mitochondrial ROSs content and an increase in glutathione levels, coupled with an enhancement in the mRNA expression of antioxidant systems (SOD1, GSR, and CAT). In addition, a significant improvement in basal oxygen consumption, maximal respiration, and ATP production was observed. These findings demonstrate that moderate weight loss can improve insulin resistance, lipid profiles and subfractions, inflammatory and oxidative stress parameters, and mitochondrial respiration. Therefore, we can affirm that dietary intervention can simultaneously achieve significant weight loss and improve metabolic profile and mitochondrial function in obesity.

## 1. Introduction

Weight loss in obesity is crucial, both for its treatment and to prevent the development of multiple obesity-related metabolic comorbidities, such as type 2 diabetes mellitus, dyslipidemia, hypertension, and metabolic syndrome [1]. Reversing obesity becomes more important in light of its association with an increased risk of developing cardiovascular disease (CVD) and mortality [2]. Adipose tissue dysfunction in the obese population is considered to be the main contributor to the development of obesity-associated comorbidities [1]. This dysregulation of adipose tissue involves, on the one hand, the production of chronic low-grade inflammation due to overproduction of proinflammatory cytokines, macrophage accumulation, adipocyte death, and endoplasmic reticulum stress. In subjects with obesity, adipose tissue releases proinflammatory cytokines and adipokines, such as TNF-α and IL-6 [3,4], and an overproduction of reactive oxygen species (ROSs), causing an imbalance in redox status and increasing oxidative stress [5]. Chronic low-grade inflammation associated with obesity and oxidative stress plays a crucial role in the development of comorbidities associated with obese population [6]. Obesity-induced oxidative stress can lead to endothelial dysfunction, characterized by impaired nitric oxide bioavailability, and increased production of vasoconstrictors; thus, excessive ROSs production can contribute to hypertension development and increase CDV risk [5,6]. In addition, the inflammatory response induced by oxidative stress can impair insulin signaling pathways, thus promoting an altered glucose metabolism [6,7]. Furthermore, inflammation and oxidative stress are closely linked processes, and both can perpetuate each other, leading to a vicious circle and facilitating the development of metabolic syndrome [6,8].

Mitochondria play a central role in the pathophysiology of obesity, during which they regulate cell metabolism, ATP production, β-oxidation of fatty acids, oxidative stress, and inflammation [7]. In the obese population, the excess production of ROSs can lead to damage to mitochondrial DNA, proteins, and lipids [5,8]. Consequently, mitochondrial enzymatic activities can be altered, including ATP generation and the antioxidant defense system, along with defects in mitochondrial biogenesis. Therefore, oxidative stress in obesity can compromise both mitochondrial function and structure [8,9]. As a result, mitochondrial dysfunction in obesity reduces substrate oxidation, causing lipid accumulation and promoting an environment overloaded with nutrients. This environment increases adhesion molecule production, contributing to greater macrophage recruitment and an excessive release of proinflammatory cytokines in adipose tissue, resulting in the perpetuation of chronic inflammation and the promotion of insulin resistance [6,9]. Likewise, elevated glucose levels in insulin-resistant individuals increase the production of ROSs, causing mitochondrial damage [7,9]. Although ROSs are naturally produced in mitochondria, their excess in obesity (compounded by a reduced antioxidant capacity and an exacerbated inflammation response) will lead to mitochondrial dysfunction [10,11,12].

Mitochondrial dysfunction in obesity involves a reduced number of mitochondria, altered biogenesis, and impaired function, all of which contribute to metabolic diseases like insulin resistance and type 2 diabetes [6,9]. There is a link between mitochondrial dysfunction and insulin resistance, with altered mitochondrial respiration and electron system being observed in obese individuals, though results vary by population and tissue [9,13,14,15,16]. Dietary interventions and nutritional supplementation can ameliorate the inflammatory response and oxidative damage in obesity [6,11]. Moreover, dietary interventions based on weight loss in the obese population have been shown to enhance the redox imbalance and the inflammatory response [17,18,19,20]. Furthermore, rapid weight loss through a very low-calorie diet (VLCD) in patients with obesity may improve both inflammation and oxidative stress parameters and enhance the antioxidant response [21,22]. Lastly, weight loss-based interventions can also improve mitochondrial respiration dysfunction, though most research shows this to be achieved through bariatric surgery rather than a low-calorie diet (LCD) [20,23,24].

Given the critical role of mitochondrial dysfunction in the development of obesity-related comorbidities, we aimed to determine if a very low-calorie diet can improve inflammation markers, oxidative stress response, and mitochondrial respiration capacity in the obese population. Therefore, the purpose of this study was to assess the effect of moderate weight loss achieved by dietary intervention on mitochondrial function and inflammatory and metabolic profiles.

## 2. Materials and Methods

### 2.1. Subjects

An interventional study was carried out in 109 subjects diagnosed with obesity. Participants were recruited among patients seeking treatment to lose weight at the Endocrinology and Nutrition Department of University Hospital Dr. Peset in Valencia (Spain).

Obese individuals (body mass index, BMI, ≥30 kg/m^2^) aged 18 to 60, with a diagnosis of obesity of at least five years and with a stable weight in the previous three months were eligible for inclusion in the study. Exclusion criteria were severe illness, history of chronic inflammatory disease, secondary obesity (untreated hypothyroidism, Cushing’s syndrome), pregnancy, and lactation.

This project was approved by the hospital’s Ethics Committee (Code: 92/18). It was conducted according to the guidelines established by the Declaration of Helsinki, and written informed consent was obtained from all participants. The study has been registered on ClinicalTrials.gov with Protocol Registration Identifier NCT06279780. 

### 2.2. Dietary Intervention

After an initial evaluation, patients underwent treatment with two cycles of a very low-calorie diet (VLCD) and 6 weeks each. Subjects consumed a liquid formula (Optisource, Nestlé S.A., Vevey, Switzerland) providing 82.2 g carbohydrates, 45.0 g protein, 13.5 g lipids, no fiber (traces), and essential vitamins and minerals based on Recommended Dietary Allowances (RDA). The energy provided by this formula was 2658 kJ per day (630 kcal per day). 

Between the two VLCD cycles, patients underwent a low-calorie diet (LCD) for 12 weeks. A registered dietician performed an individualized nutritional assessment to calculate resting energy expenditure (REE) with the aim of reducing by 500 kcal the daily caloric expenditure of each individual. REE was measured using validated REE-predictive regression equations based on the fat-free mass estimated from the impedance analysis. Subsequently, personalized hypocaloric diets adapted to the dietary habits of each patient and following macronutrient intake recommendations (50–55% carbohydrates, 15% proteins, and 30–35% fats) were prescribed. A secondary aim was to enhance fiber intake by increasing consumption of fruits, legumes, and vegetables and also prioritizing whole grain cereals. Furthermore, patients were recommended to avoid foods rich in sugars. In essence, dietary recommendations were based on improving Mediterranean diet adherence and following the dietary recommendations given by the Spanish Society of Community Nutrition. The expected effect of a VLCD was rapid weight loss. Meanwhile, in the case of the LCD, the expected effects were to both ensure progressive weight loss between VLCD phases (even if weight loss was not as great as with a VLCD) and incorporate healthy habits following the Spanish dietary guidelines. The overall objective of the dietary intervention was to achieve significant weight loss and ensure that participants could maintain it after completing the protocol with healthy dietary habits.

Dietary monitoring was conducted every 6 weeks to assess the effectiveness of the intervention and evaluate adherence through the dietary interview. Furthermore, participants were clinically monitored by an endocrinologist during the VLCD cycles. Both medical and dietary monitoring allowed us to avoid nutritional biases and the uncontrolled variability of the physiological–metabolic response to the dietary factor. It also prevented side effects of VLCD-induced rapid weight loss, such as dizziness, fatigue, and/or vitamin and mineral deficiency. Throughout the dietary intervention, participants received detailed instructions regarding the prescribed diet. A daily intake of more than two liters of calorie-free liquids was recommended. The patients’ medical prescriptions remained unaltered. 

Anthropometric parameters (weight, BMI, waist circumference), systolic (SBP) and diastolic (DBP) blood pressure, and body composition (measured through bioelectrical impedance with vector analysis seca^®^ mBCA 514/515) were evaluated at baseline and 6 months after the intervention. Waist circumference was included in nutritional assessment since it allows us to better measure the impact of weight loss on abdominal obesity in comparison with BMI alone. Likewise, vectorial analysis of body composition with impedance provides better monitoring of the evolution of the nutritional state and changes in fat mass and fat-free mass along the dietary intervention. In turn, the provided information through bioelectrical impedance allows dietary recommendations to be adapted more precisely for each participant.

### 2.3. Biochemical and Inflammatory Parameters

Venous blood samples were collected in the morning after 12 h overnight fasting at baseline and after the intervention. The following parameters were evaluated: glucose and lipid profiles, liver and renal function, nutritional status, hormone profile, blood count, and coagulation. HOMA-IR values were calculated by applying the following equation: [(fasting plasma glucose (mg/dL) × fasting serum insulin (µUI/mL))/405]. Biochemical determinations were carried out by the hospital’s Clinical Analysis Service.

The Quantimetrix Lipoprint system (Redondo Beach, CA, USA) was used to isolate LDL and HDL subfractions. High-resolution polyacrylamide gel tubes specific for both LDL and HDL were used for electrophoresis. Sequentially, twenty-five microliters of sample were mixed with 200 or 300 µL of lipoprint loading gel containing Sudan Black B dye. This mixture was placed on the upper part of the polyacrylamide gel, and electrophoresis was carried out following the manufacturer’s recommendations. Lipoprotein subfractions were quantified using a validated computerized method. 

Adipokine and proinflammatory cytokine levels were determined using the Luminex^®^ 200 analyzer system (Luminex Corporation, Austin, TX, USA). The procedure was based on the instructions provided by the MILLIPLEX^®^ Kit manufacturer (Millipore Corporation, Billerica, MA, USA). 

### 2.4. Oxidative Stress Determinations

Markers of oxidative stress were analyzed using a flow cytometry assay (Accuri C6, BD Biosciences, Franklin Lakes, NJ, USA). This assay utilized blue and red lasers (at 488 nm and 640 nm, respectively) with a 530/30 filter in FL1, 585/40 in FL2, 610/20 in FL3, and 675/25 in FL4. A total of 500 µL of whole blood was lysed using Red Blood Cell Lysis Solution (Miltenyi Biotech, Bergisch Gladbach, Germany) and then centrifuged to remove the erythrocytes. The resulting pellet was resuspended in 200 µL of HBSS and incubated with 4 µL of allophycocyanin (APC) anti-human CD45 antibody (Invitrogen, Life Technologies, OR, USA) for 20 min in the dark to label the leukocytes. Subsequently, 1:10 dilutions were prepared and incubated for 10 min with the specific fluorescence probe to be analyzed. To determine glutathione levels, we used 5-chloromethylfluorescein diacetate (5 µM, Invitrogen, Life Technologies, Eugene, OR, USA), and to measure total reactive oxidative species (ROSs) and mitochondrial ROSs, we employed 2′,7′-dichlorodihydrofluorescein diacetate (5 µM, Invitrogen, Life Technologies, OR, USA) and MitoSOX red (3 µM, Invitrogen, Life Technologies, OR, USA), respectively. Dihodroethidium (DHE) (5 µM, Invitrogen, Life Technologies, OR, USA) was used to quantify superoxide content. Lastly, we used tetramethylrhodamine methyl ester (3 µM, Invitrogen, Life Technologies, OR, USA) to determine mitochondrial membrane potential. 

Antioxidant capacity was quantified in serum by means of the e-BQC portable device (Bioquochem, Oviedo, Spain), which is a system based on measuring the redox potential in microcoulombs (µC). Samples of 100 µL were dispensed on disposable strips in order to obtain readings of rapid antioxidant (QA) and slow antioxidant (QB) responses.

### 2.5. PBMC Extraction and Real-Time Metabolic Flux Analysis

Peripheral blood mononuclear cells (PBMCs) were isolated with the MACSprepTM kit (Miltenyi Biotec, Teterow, Germany), following the manufacturer’s guidelines. Mitochondrial oxygen consumption rates (OCR) or extracellular acidification rate (ECAR) of PBMCs were measured in real time with an Agilent Seahorse XFp HS Mino Analyzer using the Seahorse XFp Cell Mito Stress Kit (Santa Clara, CA, USA), following the manufacturer’s instructions. Immediately after PBMC extraction, the pellet was resuspended with Seahorse XF DMEM medium, pH 7.4 with 5 mM HEPES, and supplemented with glucose 10 mM, pyruvate 1 mM, and glutamine 2 mM. PBMCs were seeded at a density of 3 *×* 10^5^ cells/well with final volume of 180 µL in mini culture plates that had been pretreated 24 h before with 0.1 mg/mL poly-D-lysine. Kit compounds were loaded into the injection ports of the prehydrate sensor cartridge following the manufacturer’s volume recommendations, with a final concentration of 1.5 µM oligomycin A, 1 µM carbonyl cyanide-4 (trifluoromethoxy) (FCCP), and 0.5 µM rotetone/antimycin. In response to oligomycin A, OCR should significantly decrease and ECAR should increase due to the inhibition of Complex V in the mitochondrial respiratory chain. FCCP should lead to an increase in OCR and a subsequent acidification of the extracellular medium due to mitochondrial membrane uncoupling. The injection of rotetone/antimycin inhibits Complex I and Complex III, respectively, resulting in an immediate halt of mitochondrial respiration. Multiple parameters are obtained in this one assay, including basal respiration, ATP-linked respiration, maximal and reverse capacities, and non-mitochondrial respiration.

### 2.6. RNA Extraction and RT-qPCR

RNA was extracted from PBMCs from patients with obesity—(2.5 *×* 10^6^ cells) preserved at −80 °C in RNAlater (Thermo Fisher Scientific, Waltham, MA, USA)—using the Ribospin RNA Extraction Kit (GeneAll, Seoul, Korea). RNA quantity and quality were assessed using the Nanodrop 2000 (Thermo Fisher Scientific, Waltham, MA, USA), aiming for an A260/A280 ratio of approximately 2. In brief, a total of 1000 ng of RNA was converted to cDNA using the RevertAid First Strand cDNA Synthesis Kit (Thermo Fisher Scientific, Waltham, MA, USA) under the following conditions: 5 min at 25 °C, 60 min at 42 °C, 5 min at 70 °C, and a final step at 4 °C (final volume of 20 µL). The genes of interest (CAT, GPX1, SOD1, and GSR) were amplified and quantified using the 7500 Fast Real-Time PCR System (Thermo Fisher Scientific) and FastStart Universal SYBR Green (Sigma-Aldrich, St. Louis, MO, USA). CAT, GPX1, SOD1, and GSR, and 18S (Sigma-Aldrich, St. Louis, MO, USA) and their specific sequence primers were designed as follows: CAT: forward, 5′-CTTCGACCCAAGCAACATGC-3′, and reverse, 5′-CGGTGAGTGTCAGGATAGGC-3′; GPX1: forward, 5′-TTGAGAAGTTCCTGGTGGGC-3′, and reverse, 5′-CGATGTCAGGCTCGATGTCA-3′; SOD1: forward, 5′-GGTGTGGCCGATGTGTCTAT-3′, and reverse, 5′-TTCCACCTTTGCCCAAGTCA-3′; GSR: forward, 5′-GTGGAGGTGCTGAAGTTCTCC-3′, and reverse, 5′-AACCATGCTGACTTCCAAGC-3′; 18s: forward, 5′-ACCCGTTGAACCCCATTCGTGA-3′, and reverse, 5′-GCCTCACTAAACCATCCAATCGG-3′.

The PCR conditions were as follows: 10 min at 95 °C, followed by 40 cycles of 10 s at 95 °C and 30 s at 60 °C, and, finally, two cycles of 15 s at 95 °C and 1 min at 60 °C for the melting curve (final volume of 10 µL). Samples were analyzed in duplicates, and primer sequences were designed using NCBI BlastN (https://blast.ncbi.nlm.nih.gov/Blast.cgi?PROGRAM=blastn&PAGE_TYPE=BlastSearch&LINK_LOC=blasthome accessed on 15 June 2024). Results were normalized to the expression of the housekeeping gene 18S RNA and expressed as ΔΔCt. To prevent DNAse and RNAse activity, RNAseAway (Thermo Fisher Scientific, Waltham, MA, USA) was used throughout the procedure, and no template controls were included during the qPCR process.

### 2.7. Statistical Analysis

For statistical analysis of the data, we employed the statistics program SPSS 22.0 software (SPSS Statistics IMC., Chicago, IL, USA). Continuous variables are expressed as mean ± standard deviation (SD) or as the median and interquartile range (25th–75th percentile) for parametric and non-parametric parameters, respectively. Qualitative variables are presented as percentages. The data in the figures are represented as mean ± standard error (SE). The data were examined with either a paired Student’s *t*-test or a Wilcoxon test for parametric and non-parametric data, respectively. To assess correlations between variables, a bivariate correlation with Spearman’s rho was applied. All the tests used a confidence interval (CI) of 95%, and statistical significance was considered when *p* < 0.05.

## 3. Results

### 3.1. Body Composition and Biochemical Determinations

A total of 109 individuals (64% female) within an age range of 42.3 ± 10.1 years and with a BMI of 41.0 ± 7.4 kg/m^2^ were included in the study. A total of 28% of participants had diagnosed hypertension (of which 87% were on antihypertensive treatment), 7% had type 2 diabetes (among which only 43% were being treated with antidiabetic medication), and 20% had dyslipidemia (of which 35% were receiving hypolipemiant treatment). Regarding the metabolic profile, 55% of participants had been diagnosed metabolic syndrome. A total of 6% had cardiovascular disease, of which 57% had diagnosed ischemic cardiopathy, 29% had hypertrophic cardiomyopathy, and 14% had atrial fibrillation treated with cardioversion.

After the dietary intervention, we observed an average weight loss of 11.5 ± 7.1%. This was coupled with a reduction of 19% and 32% in fat mass (FM) and visceral fat, respectively, along with a significant improvement in resistance without altering reactance. Additionally, we found a significant improvement in lipid and carbohydrate metabolism, along with a significant attenuation of both SBP and DBP (Table 1). 

Regarding the inflammatory profile of our patients, we observed a significant improvement in both hs-CRP and C3 protein levels. Furthermore, there was a significant increase in adiponectin levels after the intervention, but no improvement in resistin levels. We also observed a significant reduction in plasminogen activator inhibitor 1 (PAI-1) and intercellular adhesion molecule 1 (ICAM-1) levels, alongside a substantial increase in vascular cellular adhesion molecule-1 (VCAM-1) levels. In contrast, there was no improvement in the interleukin profile following the intervention.

At baseline, visceral fat correlated significantly with SBP (r = 0.393, *p* < 0.001), DBP (r = 0.302, *p* = 0.004), HOMA-IR (r = 0.316, *p* = 0.001), adiponectin (r = −0.273, *p* = 0.019), and PAI-1 (r = 0.289, *p* = 0.012). Concerning weight loss, significant correlations were also detected with HOMA-IR (r = −0.422, *p* < 0.001), A1c (r = −0.321, *p* = 0.001), LDL-C (r = −0.253, *p* = 0.010), and TG (r = −0.392, *p* < 0.001). Furthermore, weight loss correlated significantly with inflammatory and adhesion parameters, including resistin (r = 0.289, *p* = 0.013), PAI-1 (r = −0.301, *p* = 0.008), IL-1β (r = 0.289, *p* = 0.011), IL-10 (r = 0.263, *p* = 0.023), and VCAM-1 (r = 0.295, *p* = 0.046). 

**Figure 1:**
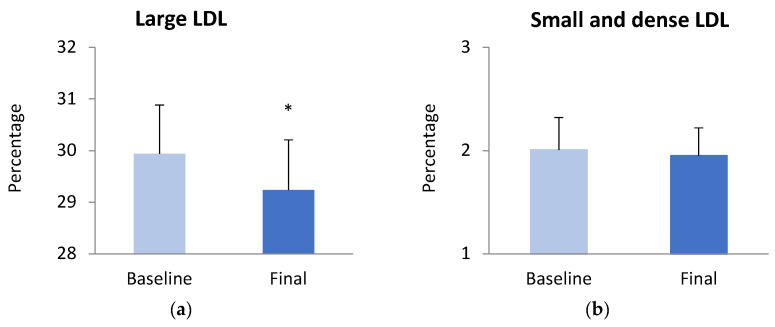

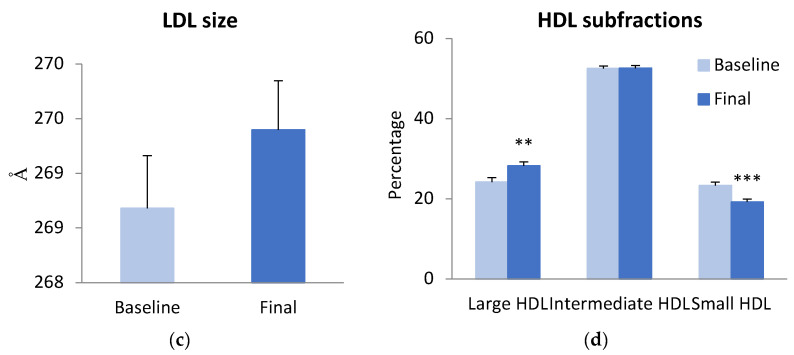
LDL-C and HDL-C subfractions in subjects with obesity before and after weight loss provoked by a dietary intervention based on a VLCD, quantified by the Quantimetrix Lipoprint system. (**a**) Large LDL-C levels and (**b**) small-and-dense LDL-C levels are expressed as percentages, respectively; (**c**) LDL-C size is expressed as Angstroms; (**d**) HDL-C subfractions are expressed as percentages. Comparisons were made using a paired Student’s *t*-test; * *p* < 0.05; ** *p* < 0.05.; *** *p* < 0.05.

When lipoprotein subclasses were analyzed, we observed a significant reduction in large LDL-C after the intervention, but similar levels in small and dense LDL-C levels, while we found no significant changes in LDL size (Figure 1). Concerning HDL-C subfractions, participants displayed a significant increase in large HDL-C coupled with a substantial reduction in small HDL-C. 

### 3.2. Oxidative Stress Parameters and Antioxidant Capacity

**Figure 2:**
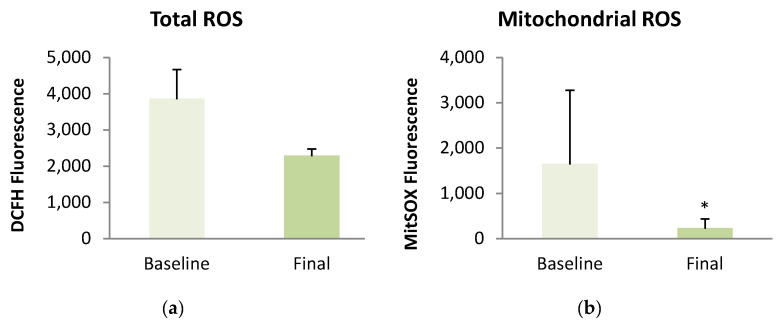

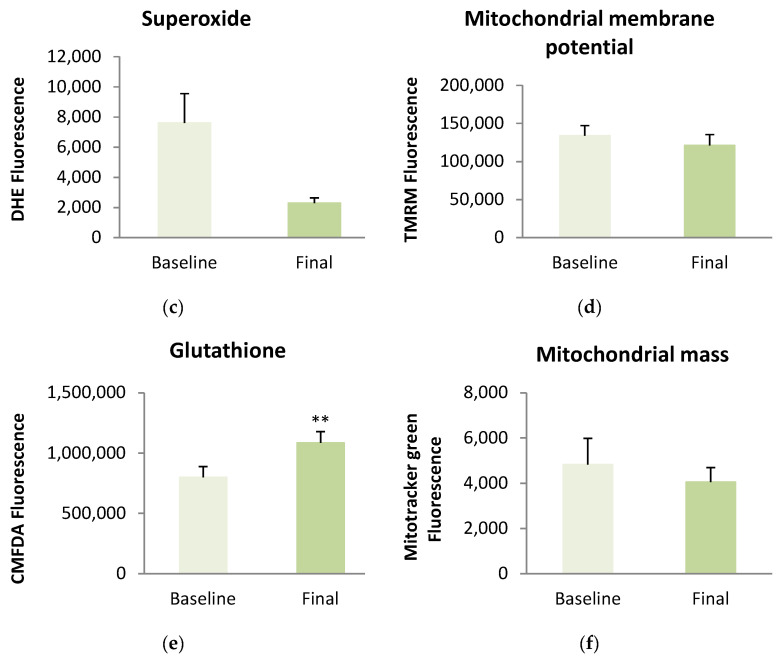
Oxidative stress and mitochondrial function parameters in the obese population before and after a very-low caloric diet. (**a**) Total ROSs; (**b**) Mitochondrial ROSs; (**c**) Superoxide; (**d**) Mitochondrial membrane potential; (**e**) Glutathione; (**f**) Mitochondrial mass. Comparisons were made using a U Mann–Whitney test; * *p* < 0.05; ** *p* < 0.01.

With respect to oxidative stress parameters after the intervention, we observed that participants presented a significant decrease in mitochondrial ROSs content along with a significant increase in glutathione levels in leukocytes. Mitochondrial mass also showed a slight improvement after the weight loss (*p* = 0.056) (Figure 2).

We found that fat mass was positively correlated with mitochondrial ROSs content (r = 0.235, *p* = 0.029) and superoxide content (r = 0.266, *p* = 0.035). Furthermore, mitochondrial membrane potential was significantly correlated with both Lp(a) (r = −0.230, *p* = 0.029) and ApoA (r = 0.318, *p* = 0.002). Additionally, glutathione content showed a significant correlation with A1c levels (r = −0.286, *p* = 0.038).

**Figure 3:**
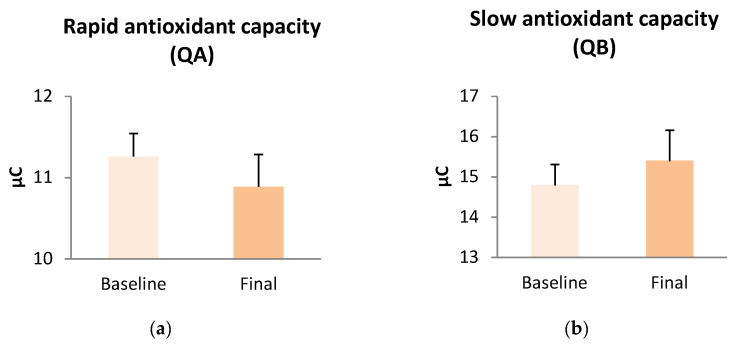
Antioxidant capacity response in subjects with obesity before and after dietary intervention. (**a**) Rapid antioxidant response (QA); (**b**) Slow antioxidant response (QB).

Concerning serum antioxidant capacity, we did not find a significant enhancement in either fast- or slow-acting antioxidants (QA and QB, respectively) after the dietary intervention (Figure 3). However, we did observe that QA correlated significantly with HOMA-IR (r = 0.327, *p* = 0.013), BMI (r = 0.379, *p* = 0.004), and visceral fat (r = 0.352, *p* = 0.007). Conversely, QB correlated significantly with HOMA-IR (r = 0.302, *p* = 0.022), IL-10 (r = 0.400, *p* = 0.011), and ICAM-1 (r = 0.599, *p* = 0.007). 

### 3.3. Measurement of Mitochondrial Respiration

**Figure 4:**
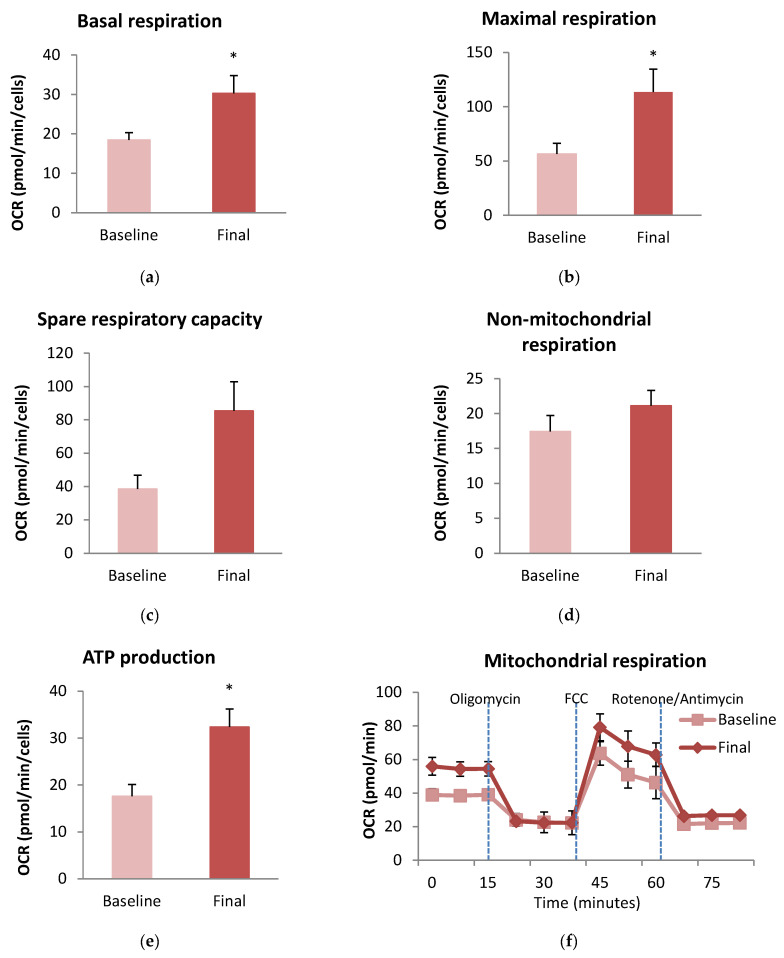
Mitochondrial function in peripheral blood mononuclear cells (PBMC) from patients with obesity before and after the dietary intervention. (**a**) Oxygen consumption rate before first compound injection; (**b**) Maximal respiration corresponds to the maximal rate of respiration that the cell can achieve after FCCP injection; (**c**) Spare respiratory capacity indicates the cell’s capacity to adapt to energy demand; (**d**) Non-mitochondrial respiration refers to non-mitochondrial oxygen consumption and indicates how much oxygen continues to be consumed by a subset of cellular enzymes even after the injection of rotenone and antimycin A; (**e**) ATP production reflects the amount of ATP generated after inhibiting mitochondrial respiration with oligomycin; (**f**) Representative basal oxygen consumption rate during the Mito stress test performed before and after the dietary intervention. Oligomycin targets ATP synthase (complex V); phenylhydrazone (FCCP) increases the enzymes involved in the electron transport chain in the inner mitochondrial membrane; and rotenone and antimycin A decrease complex I and III, respectively. Comparisons were made using a paired Student’s *t*-test (n = 18); * *p* < 0.05.

After the intervention, analysis of oxygen consumption rate during the Mito stress test revealed a similar proton leak, non-mitochondrial respiration, and coupling efficiency (Figure 4). Nevertheless, we found that the intervention significantly improved basal respiration (*p* = 0.04), maximal respiration (*p* = 0.034), and ATP production (*p* = 0.010). In addition, there was a slight improvement in spare respiratory capacity (*p* = 0.053) (Figure 4).

Remarkably, bioelectrical parameters like phase angle correlated more significantly than body weight, BMI, or visceral fat per se. Phase angle was significantly correlated with proton leak (r = 0.566, *p* = 0.014) and coupling efficiency (r = −0.640 m, *p* = 0.004). We also observed that the percentage of weight loss was negatively correlated with both basal respiration (r = −0.544, *p* = 0.029) and maximal respiration (r = −0.503, *p* = 0.047). Moreover, basal mitochondrial respiration was positively correlated with A1c levels (r = 0.485, *p* = 0.042).

### 3.4. Measurement of Antioxidant Gene Expression 

**Figure 5:**
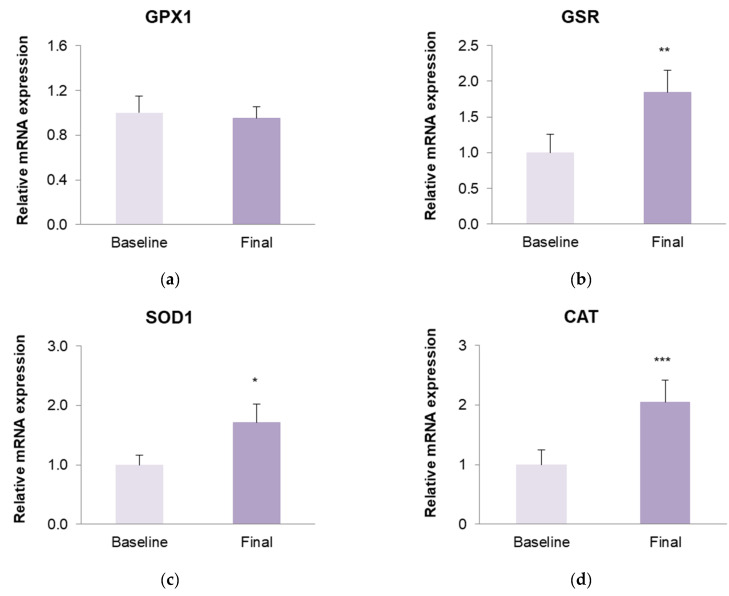
Relative mRNA expression of (**a**) GPX1; (**b**) GSR; (**c**) SOD1; (**d**) CAT on peripheral blood mononuclear cells (PBMC) from patients with obesity before and after the dietary intervention. Comparisons were made using a paired Student’s *t*-test; * *p* < 0.05; ** *p* < 0.01; *** *p* < 0.001.

We found significantly increased GSR, SOD1, and CAT mRNA levels in obese participants after dietary intervention (Figure 5). GPX1 did not show significant differences in mRNA levels after weight loss. 

## 4. Discussion

In our middle-aged obese population, the moderate weight loss achieved through a VLCD improved anthropometric and biochemical parameters. Furthermore, dietary intervention ameliorated the inflammatory response and oxidative stress, notable in a decrease in mitochondrial ROSs production and an increase in glutathione levels. In addition, an improvement in oxygen consumption rate and ATP production was observed. In this way, weight loss led to an improvement in redox balance and mitochondrial respiration, both key factors in mitochondrial dysfunction due to insulin-resistant obesity. This response was associated with an improvement in lipoprotein profile and insulin resistance, coupled with a decrease in acute-phase inflammatory proteins, such as hs-CRP and C3, and a reduction in levels of pro-atherosclerotic molecules, such as PAI-1 and ICAM-1.

The excess of adipose tissue in obesity is associated with a shift in adipokine secretion towards a pro-atherosclerotic profile, in which the adiponectin level is reduced while resistin and inflammatory cytokines levels will be steadily increased as macrophage recruitment rises [1,25]. Both CRP and C3 are acute phase reactants mainly produced in the liver, though in obesity, the excess of these proinflammatory cytokines will promote an increased synthesis of CRP and C3 in adipose tissue [26,27,28]. It has been suggested that both CRP and C3 are directly correlated with adiposity, and weight loss in an obese population could improve CRP and C3 levels, reversing the excess of adipose tissue [26,29]. Our data show that an average weight loss of 11.5% through a VLCD and LCD ameliorates the inflammatory response and improves adiponectin levels in an obese population. Weight loss through caloric restriction has been demonstrated to reduce proinflammatory mediators like hs-CRP, IL-6, and TNF-α [19,30]. In relation to the VLCD intervention in our obese population, the results are consistent with those of Kanikowska et al., who reported a significant reduction in hs-CRP levels in an obese population undergoing a VLCD [19]. However, we did not find a significant improvement in IL and TNF-α levels following weight loss, unlike other authors [22,31,32]. Strasser et al., on the other hand (and in line with our results), did not observe significant differences in IL levels in overweight and obese populations experiencing weight loss through a VLCD [33]. The effect of weight loss on serum TNF-α levels in the obese population is highly variable in the scientific literature. Several factors can increase serum TNF-α levels, such as the presence of DM2, elevated BMI, percentage of FM, and the proportion of visceral fat vs. subcutaneous fat [3,34]. Therefore, it is difficult to compare the results of different authors. López-Domènech et al. demonstrated that weight loss through a VLCD followed by an LCD significantly decreased TNF-α levels, though participants showed higher basal levels of serum TNF-α and there was a higher prevalence of DM2 in their population [22]. Kanikowska et al. also found that LCD-induced weight loss in obese participants can significantly improve serum TNF-α levels, but participants also showed higher baseline levels of serum TNF-α [19]. On the contrary, weight loss interventions through a VLCD or LCD in obese or overweight participants with lower baseline serum TNF-α levels did not reach a significant decrease despite achieving a similar percentage of weight loss [31,33]. 

The decrease in inflammatory parameters in our population was accompanied by a significant increase in adiponectin levels. This result is in line with the significant improvement in adiponectin levels in an obese population after weight loss through a VLCD reported by Lips et al. [31]. However, similar studies did not find a significant improvement in adiponectin production in obese participants after weight loss through a VLCD or LCD, despite a significant improvement in HOMA-IR values [17,19,35]. Concerning biomarkers of endothelial function, we found an improvement in PAI-1 and ICAM-1 levels after weight loss, but a significant increase in VCAM-1. Morel et al. reported a significant reduction in PAI-1 levels in obese women after weight loss achieved through a short-term VLCD [36]. Similarly, Solá et al. also found that weight loss through a VLCD followed by a low-calorie diet in an obese population was associated with a considerable reduction in PAI-1 levels [37]. Mathur et al. demonstrated in their meta-analysis that dietary-induced weight loss (with an LCD or a VLCD) can reduce adhesion molecules, including ICAM-1 and VCAM-1 [38], while other studies have published controversial results in the same sense [39]. That said, we have not found any evidence in the literature of an increase in VCAM-1 levels in subjects with obesity after diet-induced weight loss. 

In relation to lipoproteins, we found that weight loss thanks to the VCLD led to a significant reduction in large LDL-C without altering LDL-size or small-and-dense LDL-C. In contrast, we observed a significant enhancement in large HDL-C accompanied by a significant decrease in small HDL-C, despite total cHDL not showing any significant changes after the weight loss. Therefore, the intervention did not have an impact on small-and-dense LDL-C subfractions, but it did enhance CDV risk, achieving a significant amelioration in HDL-C subparticles [2,40,41]. López-Domènech et al. demonstrated that weight loss after a VLCD intervention followed by an LCD reduces small LDL-C, increases LDL-C size, and improves cHDL levels in an obese population [22]. Regarding weight loss through only LCD (with or without exercise), Dutheil et al. found significant reductions in both large and small LDL-C and small HDL-C, along with increased levels of large HDL-C [42]. Krauss et al. [43] and Bajer et al. [44] also reported a significant reduction in small HDL-C levels after LCD-induced weight loss. In fact, weight loss through bariatric surgery has also been associated with both an increase in large HDL-C levels and a reduction in small HDL-C levels [45]. Evidence shows that the effects of weight loss through caloric restriction on cHDL levels may vary depending on whether subjects are in an active weight loss phase or in maintenance after the intervention. Rolland et al. showed that VLCD in obese populations may initially decrease cHDL levels during weight loss but then return to baseline levels or even show an overall increase during weight maintenance [46]. Our population was still in active weight loss at the end of the dietary intervention; this may be the main reason why we did not find an improvement in cHDL levels despite the achieved weight loss, in contrast with similar dietary interventions. Moreover, it is not known whether being in the active weight loss phase or in maintenance might affect cHDL subfractions.

With respect to oxidative stress, evidence shows it is a key mechanism in obesity, the pathogenesis of insulin resistance, and in the development of its associated comorbidities [12]. In an obese population, oxidative response is increased as a result of an overproduction of proinflammatory mediators that promote ROSs production. In addition, the excess of free fatty acid circulation and bioavailability for oxidation—conditions typical of obesity and insulin-resistance—contribute to a rise in ROSs levels [10]. The increased production of ROSs in obesity is accompanied by a lower activity of the enzymes responsible for ROSs removal (GPx, CAT, SOD) [10,11,12]. This leads to morphological changes in mitochondria, thus contributing to mitochondrial dysfunction [7,9]. As expected, our results showed a clear, positive effect on redox status; after diet-induced weight loss, glutathione levels and mitochondrial mass improved and there was a reduction in mitochondrial ROSs. These findings are consistent with previous research, all of which demonstrated a significant improvement in the oxidative response in the obese population following weight loss through a VLCD [21,22,47,48]. In contrast, weight loss interventions consisting of an LCD rather than a VLCD may not be sufficient to achieve a significant improvement in the oxidative stress response, as observed by Asghari et al. [49] and Sofi et al. [50]. In addition to an enhancement in redox balance, we observed an increased expression of antioxidant genes (GSR, SOD1, and CAT) after VLCD-induced weight loss in our population. It is well established that obese populations show a downregulation of genes codifying antioxidant enzymes as glutathione peroxidase (GPX), superoxide dismutase (SOD), and catalase (CAT) [51]. Accordingly, obese individuals present a reduced capacity to cope with oxidative stress, contributing to an increased ROSs production, hence an increase in inflammation response [51,52]. Although some authors have observed improved activity of antioxidant enzymes after weight loss in subjects with obesity [19,22,53], few studies have assessed mRNA expression of antioxidant genes after weight loss. Merra et al. reported an enhanced expression of SOD1 after a dietary intervention based on a very low-carbohydrate and high-fat ketogenic diet in an overweight population [54]. Blum et al. observed a significant decrease in CAT expression in obese women after weight loss through bariatric surgery [55]. In contrast to Abad-Jiménez et al., we did not find increased mRNA GPX1 levels after weight loss [45]. These differences may suggest that a VLCD does not achieve similar weight loss to bariatric surgery. An improved mRNA expression of antioxidant genes does not always translate to an increased antioxidant enzymatic activity. However, the observed improvement in redox status in our population after weight loss could partly be explained by this enhancement in the expression of antioxidant genes, and thus allow for an improved mitochondrial respiration [56].

Despite the ameliorated oxidative response, we did not detect a significant improvement in antioxidant capacity after weight loss. Choromańska et al. reported a significant reduction in total antioxidant capacity (TAC) in an obese population after weight loss through bariatric surgery [57]. However, other studies based on diet-induced weight loss did not find a significant change in TAC [49,58], as observed in our population. Unfortunately, these studies cannot be easily compared due to huge differences in the type and duration of the interventions and the method used to determine TAC values. Moreover, Anaya-Moura et al. demonstrated that TAC is associated with uric acid levels in an obese population [59]. Uric acid has antioxidant properties in plasma; it can prevent lipid peroxidation, scavenge circulating free radicals, and provide up to 60% of the total antioxidant capacity [59]. Weight loss after a dietary intervention implies a decrease in uric acid levels, which may be why we did not observe a significant increase in TAC after our dietary intervention. Nevertheless, our TAC values correlate with HOMA-IR in a similar way to that reported by Choromańska et al. [57].

Mitochondria provide energy through the process of oxidative phosphorylation (OXPHOS), which generates ATP. Mitochondrial respiratory capacity refers to the ability of mitochondria to produce enough energy in order to meet cellular ATP demand [16]. Therefore, altered mitochondrial respiration can be considered a reflection of mitochondrial dysfunction in terms of bioenergetics. Decreased OXPHOS is often associated with increased mitochondrial ROSs production [16]. Likewise, an excess of mitochondrial ROSs leads to mitochondrial damage, including the complexes involved in the electron transport system, which in turn may compromise mitochondrial respiratory capacity and consequently induce a large release of ROSs [60].

Different authors have reported altered mitochondrial respiration in obese populations [14,16]. Interestingly, disorders in mitochondrial respiration are more pronounced in subjects with obesity with metabolic comorbidities than in metabolically healthy subjects with obesity. Some authors have found that obese populations with type 2 diabetes present a lower OXPHOS capacity when compared to subjects with obesity without type 2 diabetes [61,62]. Weight loss has been shown to have beneficial effects on mitochondrial function and respiration in obesity and metabolic diseases. Mechanisms of action involved would be stimulation of mitochondria biogenesis through calorie restriction-induced weight loss [63,64], reducing proinflammatory mediators with a consequent reduction in oxidative stress [5,9,16], improving insulin sensitivity [7,9], and optimizing substrate utilization [16], leading to more efficient energy production. In our population, we found a significant improvement in basal respiration, which represents an improved OXPHOS capacity. Moreover, we also observed an enhancement in both ATP production and maximal respiration, which reflects an increase in the respiratory electron-transfer-pathway (ET) capacity. This improvement mitochondrial respiration in our population would be attributable to weight loss, which has reduced inflammation response, improved insulin sensitivity, and enhanced redox response with a reduction in mitochondrial ROSs and an increase in glutathione levels. Weight loss through both bariatric surgery and exercise-based interventions in an obese population has been previously demonstrated to improve OXPHOS respiration and ET capacity in skeletal muscle fibers [23,65,66,67]. However, there are very few studies that assess the impact of diet-induced weight loss on mitochondrial respiration in subjects with obesity. Thrush et al. compared weight loss through a LCD in diet responders vs. non-responders and found an increased proton leak of muscle cells but no changes in oxygen consumption rates in responders [20]. Rabøl et al. observed a reduction in mitochondrial respiration parameters after a VLCD-induced weight loss in obese women, contrary to what might be expected [68]. The authors hypothesized that, since the samples were analyzed when weight loss was occurring and had not been stabilized, this could have been due to a metabolic adaptation to a catabolic situation. In this way, our findings in mitochondrial respiration are in line with most of the few studies based on weight loss interventions. Nevertheless, we must take into account that these results can differ depending on the tissue and type of cell analyzed, even in the same subject [69]. Measurement of mitochondrial respiration in PBMC is a less invasive option than muscle biopsy but might not correlate completely [69]. In this sense, mitochondrial respiration results between muscle cells and PBMC may not be comparable. 

With respect to glucose metabolism and its impact on mitochondrial respiration, we found a positive correlation between HbA1c levels and basal respiration. Conversely, Antoun et al. observed an inverse correlation between HbA1c and maximal uncoupled respiration [62], while Böhm et al. noted a negative correlation of mitochondrial respiration with insulin sensitivity [70]. In our population, the increasing mitochondrial respiration function in the presence of hyperglycemia could be understood as a mechanism to cope with metabolic stress. Additionally, we observed that the rate of weight loss also correlated with mitochondrial respiration, similarly to that reported by Thrush et al. [20], but no significant correlations with anthropometric parameters were found. Interestingly, bioelectrical parameters, like phase angle, showed significant correlations with mitochondrial respiration. To our knowledge, there is no study that has evaluated the relationship between bioelectrical parameters measured by vectorial impedance and mitochondrial respiration.

Indeed, diverse factors may contribute to mitochondrial dysfunction, but weight loss based on dietary interventions seems to be a first step towards reversing this situation. Few studies have assessed the effect of weight loss through caloric restriction on the expression of antioxidant genes and mitochondrial respiration in an obese population. Accordingly, our findings confirm that VLCD can be considered a useful method to achieve rapid weight loss, and to enhance both inflammatory response and redox status. Consequently, this translates into an improved mitochondrial function, reflected in mitochondrial respiration. The present research has some limitations. The first of these is the use of PBMCs instead of muscle biopsies to measure mitochondrial bioenergetics; muscle is a high energy consumer in comparison, and the results of muscle biopsies would be more reliable. Secondly, adherence to diet among our cohort was quite variable, particularly in the hypocaloric diet, between the VLCD phases. Thirdly, we have not determined the activity of the evaluated antioxidant enzymes; thus, we cannot confirm that the increased expression in mRNA is equivalent to an enhancement in the antioxidant system. Lastly, we did not assess other factors that can modulate inflammation, oxidative stress, and mitochondrial respiration, such as exercise or medication. On the other hand, the strengths of this study lie in its interventional study design with a long-term follow-up and significant weight loss despite the absence of surgery or drugs for weight loss. One of the most noteworthy findings of our study is the association of bioelectrical values with mitochondrial dysfunction parameters.

## 5. Conclusions

Weight loss through a VLCD caloric restriction is an effective strategy for reducing CDV risk, as it ameliorates the lipid profile, insulin resistance, and inflammatory response in subjects with obesity. Consequently, there is an improvement in both redox status and mitochondrial respiration in this population; both are a reflection of mitochondrial function. These findings endorse the effectiveness of calorie restriction, not only as a means of losing weight in obesity and reducing CDV risk, but also as a way of reversing mitochondrial dysfunction. Thus, diet-induced weight loss may prevent the development of obesity-associated comorbidities mediated by insulin resistance, such as type 2 diabetes mellitus.

## Figures and Tables

**Table 1:** Body composition, metabolic, and inflammatory parameters in obese participants before and after a dietary intervention based on a very low-calorie diet (VLCD).

	Baseline	Final
N (females %)	109 (64.2)	-
Age (years)	42.3 ± 10.1	-
Weight (kg)	114.4 ± 23.1	100.9 ± 21.5 ***
BMI (kg/m^2^)	41.0 ± 7.4	36.4 ± 7.2 ***
Waist (cm)	119.0 ± 16.4	110.3 ± 16.2 ***
Fat mass index (kg/m^2^)	19.7 ± 5.4	16.3 ± 5.5 ***
Visceral fat (L)	4.5 (3.2; 7.5)	3.0 (2.0; 4.6) ***
Resistance (Ω)	518.4 ± 74.1	542.7 ± 81.0 ***
Reactance (Ω)	49.6 ± 8.3	50.6 ± 8.8
Phase angle (°)	5.4 (5.1; 5.7)	5.3 (5.0; 5.6) ***
SBP (mmHg)	130.7 ± 15.2	120.6 ± 14.8 ***
DBP (mmHg)	80.7 ± 10.1	76.0 ± 9.5 ***
Glucose (mg/dL)	101.0 ± 24.5	92.3 ± 11.7 ***
Insulin (μUI/mL)	17.9 (12.4; 26.3)	11.3 (8.0; 17.7) ***
HOMA-IR	4.6 (3.0; 6.5)	2.5 (1.8; 4.1) ***
A1c (%)	5.6 ± 0.7	5.4 ± 0.4 ***
TC (mg/dL)	191.3 ± 38.1	177.7 ± 44.2 ***
HDL-C (mg/dL)	45.8 ± 10.6	45.6 ± 11.3
LDL-C (mg/dL)	118.8 ± 31.2	113.5 ± 34.7 *
TG (mg/dL)	118.0 (89.0; 166.0)	86.0 (69.5; 119.0) ***
Apo A1 (mg/dL)	139.7 ± 26.9	131.0 ± 29.7 **
Apo B (mg/dL)	101.8 ± 26.7	92.1 ± 28.5 ***
Lp(a) (mg/dL)	11.0 (4.3; 32.0)	13.5 (5.0; 29.0)
Uric acid (mg/dL)	5.6 (4.9; 6.7)	5.1 (4.3; 6.1) ***
hs-CRP (g/dL)	7.6 (4.4; 14.0)	5.1 (3.1; 11.4) ***
C3 Protein (mg/dL)	140.6 ± 20.2	128.0 ± 19.5 ***
Adiponectin (μg/mL)	19,656.9 (11,644.0; 30,243.7)	22,745.0 (16,040.8; 33,665.8) ***
Resistin (μg/mL)	26,178.1 (20,102.2; 42,631.0)	29,301.5 (22,299.5; 40,632.2)
PAI-1 (ng/mL)	123.3 ± 50.7	103.5 ± 35.4 ***
ICAM-1 (ng/mL)	143.7 ± 47.0	132.8 ± 41.7 *
VCAM-1 (ng/mL)	853.3 ± 276.0	912.1 ± 274.6 **
IL-1β (pg/mL)	1.6 (0.9; 2.6)	1.3 (0.8; 2.5)
IL-6 (pg/mL)	0.5 (0.2; 2.4)	0.3 (0.2; 1.6)
IL-10 (pg/mL)	13.4 (2.2; 31.6)	10.7 (2.4; 29.8)
TNF-α (pg/mL)	12.3 (10.7; 15.6)	12.2 (10.3; 15.4)

Data are presented as mean ± SD for parametric data, and median (IQ range) for non-parametric parameters. * *p* < 0.05; ** *p* < 0.01; *** *p* < 0.001 when compared with a paired Student’s *t*-test or U Mann–Whitney (parametric and non-parametric parameters, respectively). BMI: body mass index; SBP: systolic blood pressure; DBP: diastolic blood pressure; HOMA-IR: insulin resistance index; A1c: hemoglobin A1c; TC: total cholesterol; HDL-C: high-density lipoprotein cholesterol; LDL-C: low-density lipoprotein cholesterol; TG: triglycerides; Apo A1: apolipoprotein A1; Apo B: apolipoprotein B; Lp(a): lipoprotein(a); hs-CRP: high-sensitivity C-reactive protein; PAI-1: plasminogen activator inhibitor-1; ICAM-1: intercellular adhesion molecule 1; VCAM-1: vascular cell adhesion protein 1; IL: interleukin; TNF-α: tumor necrosis factor-alpha.

## Data Availability

The data presented in this study are available upon request from the corresponding authors. The data are not publicly available due to ethical reasons.
